# Rethinking knowledge systems for agroforestry: Insights from the mental models of cacao farmers in Colombia

**DOI:** 10.1007/s13280-025-02189-7

**Published:** 2025-04-30

**Authors:** Tatiana Rodríguez, Michelle Bonatti, Katharina Löhr, Stefan Sieber

**Affiliations:** 1 Department of Environmental Politics, Helmholtz Centre for Environmental Research – UFZ, Permoserstraße 15, 04318 Leipzig, Germany; 2 Sustainable Land Use in Developing Countries, Leibniz Centre for Agricultural Landscape Research (ZALF), Eberswalder Straße 84, 15374 Müncheberg, Germany; 3 Department of Agricultural Economics, Humboldt University of Berlin, Invalidenstraße 42, 10115 Berlin, Germany; 4 University for Sustainable Development (HNE Eberswalde), Schicklerstraße 5, 16225 Eberswalde, Germany

**Keywords:** Agricultural innovation, Cognitive mapping, Mental models, Social learning, Systems thinking

## Abstract

Agroforestry is a sustainable land-use strategy that requires better integration of diverse knowledge systems. This paper examines how formal knowledge systems for cacao agroforestry systems in Colombia are structured, and how farmers understand and manage these systems. We interviewed 42 actors involved in knowledge production and dissemination and worked with 18 farmers to create mental maps of their management practices. Our findings show that formal knowledge systems remain predominantly top-down and scientific-based, despite increasing recognition of local knowledge. Farmers’ mental models reflect this, showing reliance on external advice and simplified cause-and-effect thinking. When theoretically pooling farmers’ perspectives, we observe a more comprehensive understanding of agroforestry, suggesting that social learning can strengthen their independence and adaptive capacity. Moreover, farm management is shaped by context-dependent variables such as climate seasonality and market access, thus highlighting the importance of integrating farmers’ experiential knowledge into agroforestry design and dissemination for more sustainable, context-sensitive decision-making.

**Supplementary Information:**

The online version contains supplementary material available at 10.1007/s13280-025-02189-7.

## Introduction

Industrial agriculture, characterized by large-scale monocultures complete with the intensive use of synthetic inputs and water, is driving soil degradation, greenhouse gas emissions, and loss of agrobiodiversity (Kremen et al. 2012). At the same time, it falls short of ensuring food and nutrition security (DeLonge and Basche 2017; McGreevy et al. 2022). Transitioning toward sustainable agriculture would benefit from agroecological approaches like agroforestry systems (AFS), which integrate trees with crops and/or livestock to enhance ecological synergies, while supporting both climate change mitigation and adaptation (HLPE 2019; Gassner and Dobie 2022). AFS are increasingly recognized as knowledge-intensive, with access to knowledge being recognized as a major barrier to its uptake (Pérez-Marulanda et al. 2025). However, its inherent diversity and dynamism challenges conventional thinking on agricultural technology adoption and scaling (Halbrendt et al. 2014; Plieninger et al. 2020; Rodríguez et al. 2023).

Expanding AFS requires moving beyond the simplification of producing and disseminating prescriptive technological packages through technical advice (Coe et al. 2014; Gardeazabal et al. 2021) toward systemic changes (Global Alliance for the Future of Food 2021) in the processes of vertical scaling (policies, markets, and institutions) and horizontal scaling (geographical spread of practices) (Moore et al. 2015; Lam et al. 2020). Leveraging pluralistic knowledge systems is cross-cutting in this expansion to enable contextually relevant solutions and avoid unintended detrimental consequences (Brock et al. 2024). Here, knowledge systems are defined as sets of social and institutional arrangements for producing, disseminating, and applying knowledge (Cornell et al. 2013).

At the level of individual actors in agriculture (e.g., researchers, practitioners, technicians and farmers), knowledge systems approaches are reflected in their mental models, which can be represented graphically as cognitive maps. A map can be simple, such as a linear chain of causalities, or a more complex network of interconnected ideas, such as feedback loops or bidirectional effects showing interdependencies and synergies (Özesmi and Özesmi 2004; Levy et al. 2018). Lalani et al. (2021) find that more complex mental models among Mozambican farmers are associated with higher adoption of conservation agriculture (CA) practices and a positive perception of their benefits. Levy et al. (2018) reveal that complex causal structures are relatively uncommon among sustainable agriculture leaders in California, which could have negative implications for decision-making by not anticipating unintended consequences and not finding common ground for cooperation across knowledge systems. Therefore, emphasis should be placed on whether and how knowledge systems facilitate the development of more complex systems thinking about sustainable agriculture (Levy et al. 2018; Lalani et al. 2021). Post-rationalist perspectives on knowledge that advocate for situated social learning and actively integrate diverse forms of knowing, could help to develop more complex mental models while paving the way for the emergence of integrated solutions given contextual specificities (Ferguson et al. 2010; Norström et al. 2020a, b; Gardeazabal et al. 2021).

Further, fine-scale variation in the diverse socioeconomic and ecological contexts where AFS are promoted creates a need for local adaptation (Coe et al. 2014), but detailed local science-based data are usually lacking (Özesmi and Özesmi 2004; Mehryar et al. 2017). Therefore, much could be learned from incorporating local knowledge systems (Coe et al. 2014; Halbrendt et al. 2014; Jacobi et al. 2017; Gardeazabal et al. 2021), which comprise dynamic and complex bodies of know-how, practices and skills, all developed over time on the basis of local people’s experiences in their environmental and socioeconomic realities (Beckford and Barker 2007; Šūmane et al. 2018). Isaac et al. (2009) find that the use of local knowledge in AFS management in Ghana was critical for farm development. Baynes et al. (2011) note that without knowledge about farmers’ mental models, program-led AFS innovations may fail.

Finally, more open and inclusive knowledge systems are needed to adaptively manage social–ecological systems (Van Kerkhoff 2014; Norström et al. 2020a, b; Brock et al. 2024). Halbrendt et al. (2014) and Averbuch et al. (2022) both note not just the differences of mental models on sustainable agriculture among farmers but also between them and researchers and practitioners. Olazabal et al. (2018) find evidence of diverging and even contradictory mental models of climate impacts and potential solutions. Their findings also illustrate how integrating these differing perspectives can foster the emergence of new knowledge. Thus, more attention should be paid to the rules and norms governing knowledge systems and the power structures underlying them. Although complex systems thinking, integration of local knowledge, and open knowledge systems are recognized as crucial for addressing unsustainable agriculture, a significant research gap exists regarding how formal knowledge systems influence farmers’ cognitive frameworks. This paper addresses this gap by examining the relationship between formal knowledge systems (research, extension, and advisory services that produce, organize, and/or disseminate scientifically explicit knowledge) and farmers’ mental models for sustainable AFS management. Using cognitive mapping to analyze cacao agroforestry systems (CAFS) in Colombia, we investigate farmers’ conceptual understanding of AFS management. Although knowledge barriers to AFS adoption are documented, critical reflection on effective knowledge production and dissemination strategies remains underdeveloped.

We apply a comparative case study between two municipalities, acknowledging the need for AFS to be adapted to the diverse local contexts and also recognizing that farmers’ mental models are shaped by local conditions. Three main research questions guide this study: (1) How do formal knowledge systems in Colombia shape the production and dissemination of knowledge on cacao agroforestry?; (2) How do individual and social structures of farmers’ mental models differ across municipalities and to what extent do they address the problem of managing CAFS sustainably?; and (3) To what extent do the formal research, extension and technical advisory services contribute to more open and inclusive knowledge systems for managing CAFS?

## Analytical framework

Current agricultural knowledge systems exhibit a network of actors with diverse knowledge and expertise, and multiple learning pathways such as experiential, technical, and social (Lubell et al. 2014). Thus, agricultural knowledge systems aiming at the transition toward sustainability should seek to synergistically leverage this knowledge pluralism, generating local capacity building and empowering farmers (Ferguson et al. 2010; Norström et al. 2020a, b; Brock et al. 2024). Our analytical framework (Table 1) is based on the rationalist and post-rationalist knowledge management approaches of Ferguson et al. (2010), the approaches for creating synergies between knowledge systems of Tengö et al. (2014), and the mutually reinforcing learning pathways for agricultural knowledge systems of Lubell et al. (2014).

**Table 1:** Analytical framework for agroforestry knowledge approaches

	Rationalist approaches	Post-rationalist approaches
Knowledge epistemologies	*Objectivist approach* Knowledge as an "universal truth"	*Practice-based view* Knowledge emerges from socially constructed practices
Institutional purposes	*Knowledge transfer* Instrument delivered unchanged to solve a problem	*Situated learning* Synergies among knowledge systems embedded within a particular context
Implementation strategies	*Engineering approach* Manage and control of knowledge resources Mainly scientific explicit knowledge Technical learning pathway	*Emergent approach* Facilitating knowledge flows (tacit, explicit) within and between knowledge systems (knowledge integration, cross-fertilization, co-production) Experiential and social learning pathways
Effects/outcomes	*Domination/Hierarchy of knowledge* Marginalization of non-scientific knowledge systems Dependency on knowledge resources Prescriptive solutions Simple causal reasoning	*Democratization of knowledge* Local capacity building Farmer empowerment Integrated, more applicable, and context-sensitive solutions Complex causal reasoning

### Epistemologies guiding AFS knowledge systems

For this study, we followed the two main epistemological views on knowledge described by Ferguson et al. (2010): *objectivist* and *practice-based.* An objectivist view sees knowledge as an explicit and unchanged resource that can be transferred between a sender and a receiver to deliver a solution. Given that sustainability challenges are “wicked problems,” no objective solutions can be developed. In addressing these challenges, a practice-based epistemology reconfigures the nature of knowledge as an emergence of socially constructed practices (Ferguson et al. 2010; West et al. 2019).

### Purposes of AFS knowledge systems

Objectivist knowledge systems assume that farmers lack certain knowledge, thus positioning scientists as problem-solvers who generate and transfer solution through extension agents (Wood et al. 2014). If *knowledge transfer* is the purpose, there is a high risk of neglecting local knowledge (Ferguson et al. 2010) that is essential for adapting sustainable systems like AFS to local conditions (Coe et al. 2014). Conversely, *practice-based* approaches integrate *situated knowledge*, i.e., locally embedded knowledge, which is proven critical for promoting diverse AFS options tailored to farmers’ needs (Haggar et al. 2001; Dumont et al. 2021), thus empowering them to better cope with new challenges emerging from uncertain environments (Jacobi et al. 2017).

### Implementation strategies for AFS knowledge systems

If knowledge is perceived as an instrument, then implementation strategies focus on scientific knowledge production and dissemination typically using the technical learning pathway (Lubell et al. 2014). This pathway manages and controls knowledge resources, ensuring monitoring and replication, i.e., *an engineering perspective* (Ferguson et al. 2010). However, if knowledge is perceived as culturally embedded and practice-based, this implies implementation strategies that activate the experiential and social learning pathways; these aim at facilitating knowledge flows, and establishing generative forms of inquiry within which new and more useful knowledge can emerge, i.e., *an emergent perspective* (Ferguson et al. 2010; West et al. 2019). Tengö et al. (2014) distinguish three ways of connecting knowledge systems: (i)* integration*, which implies incorporating components of one knowledge system into another through validation processes based on the latter system; (ii)* cross-fertilization,* which emphasizes complementarities while presupposing validation across knowledge systems; *and *(iii)* co-production of knowledge*, which entails engaging in mutual processes of knowledge production at all stages. As suggested by Turnhout et al., (2020), underpinning these efforts is an ethic of mutuality, reciprocity, and equality between scientific and other knowledge systems.

### Outcomes from AFS knowledge systems

The rationalist perspective of knowledge enables technological and social change but also marginalizes other knowledge systems (de Sousa Santos et al. 2008). Instead, AFS knowledge approaches should advocate for situated social learning, thus actively and reciprocally integrating diverse forms of knowing, paving the way for the emergence of context-specific agricultural solutions, while strengthening local capacities (Ferguson et al. 2010; Norström et al. 2020a, b; Gardeazabal et al. 2021). However, without addressing political and power dimensions, these integrative efforts risk inadvertently reinforcing rationalist approaches (Turnhout et al. 2020).

Farmers’ mental models shape their decision-making processes (Halbrendt et al. 2014; Garini et al. 2017) and can be represented through cognitive maps, which illustrate their knowledge structures as networks of variables and their interconnections. Variables can be physical quantities (like precipitation), socioeconomic concepts (like income), or complex and abstract ideas (like social justice) (Özesmi and Özesmi 2004; Isaac et al. 2009; Levy et al. 2018; Averbuch et al. 2022). Cognitive mapping provides a grounded method for organizing system components and assessing whether their relationships suggest complex versus simple causal thinking, or hierarchical versus democratic system understanding (Özesmi and Özesmi 2004; Isaac et al. 2009; Levy et al. 2018). Thus, representing and analyzing farmers’ cognitive maps of CAFS knowledge can reveal the complexity of their cause-and-effect thinking, their level of empowerment, and their local capacities and practices to manage such systems. 

## Materials and methods

### Case studies description

In Colombia, cacao (*Theobroma cacao *L.) is traditionally grown by smallholder farmers within “cacao agroforestry systems” (CAFS) (Cerda et al. 2014; Rodríguez et al. 2023). These systems vary depending on biophysical conditions and household needs, and typically include a diverse mix of musaceous plants, fruit trees, leguminous species, and native woody trees. Diversification within AFS has been shown to reduce farmers’ vulnerability to climate change (Beltrán-Tolosa et al. 2022), enhance food and nutrition security (Jemal et al. 2021), support biodiversity conservation (Palacios-Bucheli and Bokelmann 2017), and offer opportunities for ecological restoration (De Leijster et al. 2021).

Given large consumption in Colombia, cacao production is largely oriented toward domestic consumption. various governmental, non-governmental, and international development organizations have promoted CAFS as a strategy for rural development. These initiatives aim to revitalize low-yielding cacao crops, establish new agroforestry systems, and provide alternatives to illicit crops or expansion of the agricultural frontier (Charry et al. 2017; Furumo and Lambin 2020).

This study compares two municipalities where such interventions have taken place: Belén de los Andaquíes (hereinafter Belén) in Caquetá and La Paz in Cesar. Belén is located in the Andean-Amazonian transition zone and has a tropical rainforest climate, while La Paz lies in the Caribbean region and experiences a tropical dry forest climate. Seasonal rainfall patterns differ markedly: La Paz is characterized by extended dry seasons and short, intense rainy periods, whereas Belén experiences longer and more intense rainy seasons. These climatic conditions impact cacao flowering and development in both areas, with the rainy season increasing pest pressures due to higher humidity.

Cacao commercialization and access to knowledge also differ substantially between the two municipalities. In La Paz, cacao is the main income source for most CAFS farmers, and the region benefits from proximity to Colombia’s major cacao-producing areas. Farmers here have better access to markets and technical support. They typically sell wet cacao beans to centralized facilities for fermentation and drying, with the final product sold domestically at higher prices when post-harvest practices are done properly. The Colombian Agricultural Research Corporation (AGROSAVIA) maintains a research center nearby, and the National Federation of Cacao Producers (FEDECACAO) has a regional office that plays a central role in producing and disseminating knowledge related to CAFS, including plant material and agroforestry arrangements.

In contrast, Belén is more remote and less connected to cacao-specific institutions. While the region has a stronger presence of farmer associations and support from various governmental and non-governmental actors, these often serve multiple agricultural sectors, including livestock and coffee. Cacao is usually sold to farmer associations following on-farm fermentation and drying, as transport is easier when dry (Rodríguez et al. 2023). Due to the influence of external projects, markets for organic cacao exports have opened up there.

Ecological and socioeconomic differences shape the composition and structure of CAFS in each municipality. In La Paz, systems typically feature banana or plantain (*Musa* spp.), malanga root (*Xanthosoma sagittifolium*), or leguminous species such as *Gliricidia sepium* for temporary shade during the early years. They also include fruit trees like avocado (*Persea americana*), citrus (*Citrus* spp.), zapote (*Matisia cordata*), and timber species like *Cedrela odorata* or *Handroanthus chrysanthus* for permanent shade. In Belén, the composition often includes banana or plantain (*Musa spp.*) and cassava (*Manihot esculenta*) as temporary shade, a wider range of fruit trees such as arazá (*Eugenia stipitata*), caimo (*Pouteria caimito*), copoazú (*Theobroma grandiflorum*), and native palms like asaí (*Euterpe precatoria*) or chontaduro (*Bactris gasipaes*). Woody and forest trees for permanent shade include *Minquartia guianensis*, *Caryodendron orinocense*, *Hieronyma alchorneoides*, and *Cedrela odorata*.

### Data collection

Data collection comprised three stages. First, we conducted semi-structured interviews with representatives of institutions providing technical advisory, extension or research for CAFS farmers in the regions where the two municipalities are located (Caquetá and Cesar), using purposive sampling method. Interviews, held in Spanish (in-person or via video call) between November 2020 and February 2021. The questions explore institutional purposes and strategies for prioritizing CAFS research needs, conducting research, and disseminating knowledge. We interviewed 15 people from Caquetá and 18 from Cesar (Supplementary Information, Table S1).

To better understand national-level policies and institutions shaping CAFS knowledge approaches, the second stage involved in-depth interviews with people leading CAFS research and technical advisory/extension services in cacao-related private and public institutions. Between April and November 2021, we interviewed nine experts in Spanish online and personally (Supplementary Information, Table S2). Participants were purposely selected based on their proven experience in research, extension, or technical advisory for cacao farming at management or operational levels and their willingness to participate. Interviews, averaging 90 min, covered topics such as CAFS research and extension strategies, national regulations, funding, effectiveness indicators, farmer participation, and their perceptions about improvement opportunities.

Third, in February 2022, we conducted in-depth interviews with a subset of 8 farmers from a CAFS association in Belén (40 members) and 10 from a CAFS association in La Paz (60 members). Farmers were selected based on specific criteria: they were adults managing their own cacao agroforestry farm for at least 3 years, belonged to a farmer association, and had access to extension services (Supplementary Information, Table S3). Due to time, budget, and accessibility constraints, we used stratified purposive sampling, dividing cacao production areas into strata (sub-zones) and selecting a purposeful sample from each sub-zone to capture diverse agroecological and socioeconomic conditions (Sandelowski 2000; Palinkas et al. 2015) All interviews were conducted in Spanish and lasted between 45 and 80 min. These interviews and those of the previous stages were recorded with interviewee consent.

**Fig. 1:**
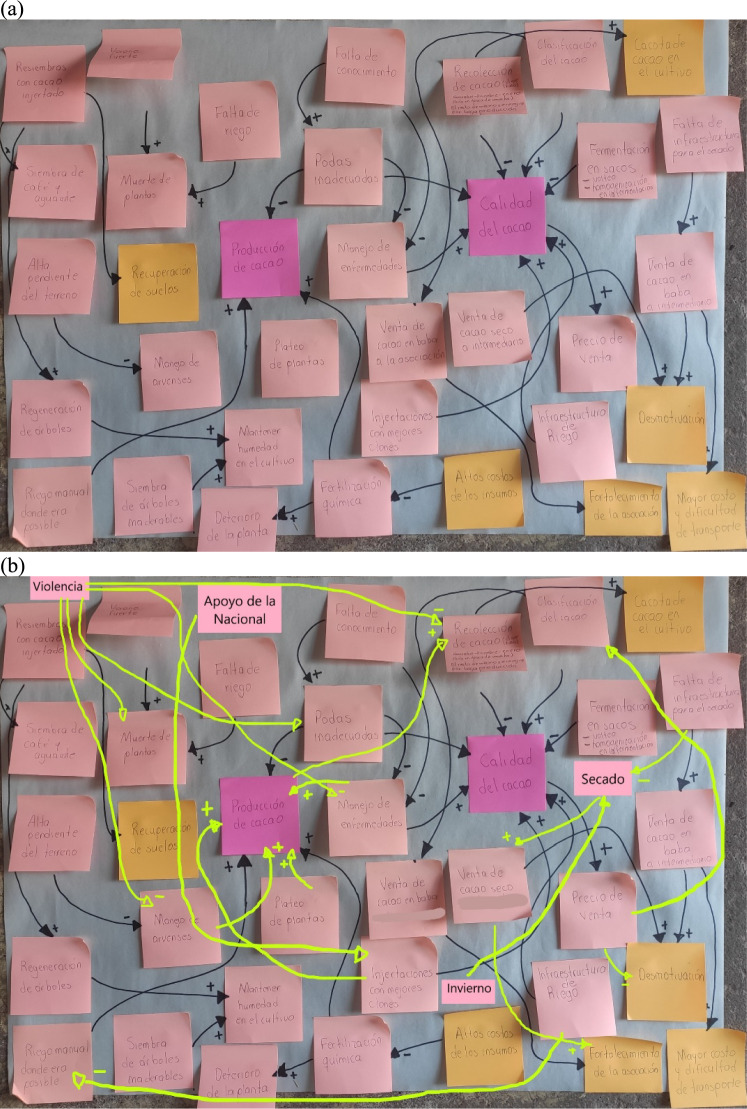
**a** Example of the cognitive map resulting from interview 16 in La Paz (Cesar) on February 19, 2022. **b** Example of the refined cognitive map after adding missing variables and connections (yellow arrows) based on the interview transcripts. Colored post-its with text represent variables mentioned by individual farmers about CAFS management on their farms. Directed arrows represent relationships between variables, “ + ” indicated positive relationships, and “-” indicated those negative relationships

Following the methodological guidelines of Özesmi and Özesmi (2004), these interviews allowed drawing individual cognitive maps (ICMs) with farmers, illustrating key variables and their relationships influencing their decision-making in CAFS management across pre-harvest, maintenance, harvest, post-harvest, processing, and commercialization. The mapping process began with two anchor nodes: land preparation for planting and product consumption or sale, which were written on post-it notes and placed on opposite sides of a poster board (Fig. 1a). The interviewer added in-between steps mentioned by farmers, as additional nodes, also on post-it notes. Once all steps were registered, farmers were asked to identify key variables influencing each step. These questions included, but were not limited to:

- What are the important factors when establishing cacao on your farm? What difficulties have you experienced while doing so?
- Have you planted other crops or woody trees along with cacao? What are the important factors when planting them? What difficulties have you encountered?
- What are important factors when weeding, pruning, fertilizing, and managing pests in your cacao production system? How do you weed, prune, fertilize, and manage pests in your cacao production system? What difficulties have you experienced?
- What are the important factors when carrying out post-harvest processes? How do you ferment and dry cacao? Do you transform cacao into another product? Have you had any difficulties in carrying out post-harvest processes?
- Which steps/factors are the most important for having good cacao production and quality?

While the interviewee was responding, the interviewer was writing variables on post-it notes and drawing the indicated relationships between them with arrowheads and values of − 1 for negative relationship and 1 for positive relationship. To complete the ICM, additional questions were asked to identify more relationships between variables. Figure 1a shows a full map after the interview was completed.

### Data analysis

Responses from the semi-structured interviews with cacao-related professionals were compiled in an Excel sheet, while in-depth interviews were fully transcribed. Both data sets were imported into MAXQDA22 and coded into four predetermined categories (knowledge epistemology, institutional purposes, implementation strategies, and outcomes) aligned with the analytical framework.

Interviews with cacao farmers were transcribed and analyzed to refine the ICMs by adding missing variables and connections (Fig. 1b). For quantitative analysis, some variables were merged and common terminology was defined (Mehryar et al. 2017), e.g., “winter” and “rains” were named as “rainy season.”

After creating all ICMs, each was transformed into an adjacency matrix. Specifically, the structure is determined by listing all concepts along the horizontal and vertical axes, then placing the connections in the intersecting cell with values of − 1 (negative relationship), 0 (no relationship), and 1 (positive relationship).

ICM structures were analyzed using graph theory indices, including the number of variables, the number of connections, the number of transmitter (with only outgoing connections), receiver (with only incoming connections), and ordinary variables (with both outgoing and incoming connections). Previous studies (Özesmi and Özesmi 2004; Isaac et al. 2009; Averbuch et al. 2022) interpret transmitters as external drivers or inputs; receivers variables as systems outcomes or implications, and ordinary variables as means or management practices. Further, we calculated the ratio of connections to variables, density, and the receiver-to-transmitter ratio to assess mental model complexity and hierarchization. ICMs indices were averaged by municipality and compared to identify differences between farmers’ mental models.

Seeking to examine the benefits of connecting farmers’ knowledge, ICMs were additively superimposed using matrix addition to create a social cognitive maps (SCMs) for each municipality (Özesmi and Özesmi 2004). All ICMs were weighted equally and the values of each connection were normalized in the summed matrix by the number of ICMs in order to put the range of the relationships between − 1 and + 1. When conflicting connections with opposite signs appeared, fuzzy causal relationship theory was applied (Kosko 1986; Kim and Lee 1998) to avoid changing the system behavior. To do so, the variable with conflicting connections is redefined by creating two variables, one with negative sense and the other with positive sense. The same graph theory indices were calculated for the two resulting SCMs and compared. Calculations were obtained in RStudio using the FCMapper package.

To compare the structures of ICMs and SCMs, we examined the most mentioned variables in the ICMs and the most central variables in each SCMs by type (transmitter, ordinary or receiver) in each municipality. This helped identify similarities and differences in the key variables that farmers consider for managing CAFS locally. Centrality, measured by cumulative indegree (incoming effects) and outdegree (outgoing effects) indicates how connected a variable is to others and the cumulative strength of those connections (Averbuch et al. 2022).

## Results

### Characterizing CAFS formal knowledge systems

The institutional purposes and implementation strategies acknowledged by the interviewees relate to their epistemology. While one expert in rural innovation and extension noted that Colombia had conceptually moved beyond an objectivist view of knowledge, others (e.g., CO2, CO3) still described knowledge as an explicit resource to be transferred. However, Interviewee CO6 recognized the complexity of CAFS management and emphasized the importance of socially constructed practices for improving research and extension services.

Most interviewees at the national and regional levels used the terms technology or knowledge transfer, emphasizing the purposes of developing and transferring technological solutions aimed at making cacao farmers more productive and competitive. While many interviewees acknowledged the importance of being sensitive to local knowledge and needs, few explicitly emphasized situated learning as part of their purposes. For example, interviewees CA6 and CE6 underlined the importance of local knowledge in tailoring the contents of technical advisory to sites.

Implementation strategies for CAFS formal knowledge systems primarily follow an engineering-driven perspective. Research and extension activities are mainly financed by a parafiscal fund managed by FEDECACAO, to which cacao farmers contribute. Additional investments come from private processing companies, research institutions, and universities. Regionally, producer associations and NGOs provide extension services using their own budgets or international cooperation funding. Research efforts focus on experimental designs at institutional facilities or on farmers’ plots to evaluate cacao clones for productivity, sexual compatibility, organoleptic quality, and pest and disease incidence. Evaluations are also made of fruit or woody species that can synergistically accompany cacao. While some strategies incorporate local knowledge, such as *participatory varietal selection* (CO5 and CO6), they must undergo a validation process based on the scientific knowledge system. New plant material requires governmental registration, while extending its use to other regions demands additional long-term testing. The registration process can take up to seven years (CO8) and institutions can only spread registered plant materials. Cacao-related social research is rarely mentioned in the interviews, with only some regional universities addressing rural sociology and extension innovation. Research results are disseminated via *“conferences, symposiums, forums, technical talks, congresses, workshops, meetings, seminars, …, delivery of planting materials, as well as fairs and visits to the research center” (CO3).* Other knowledge-sharing methodologies include diploma courses for technical assistants and extension agents (CA11) alongside audiovisual material shared on social networks (CE9, CE10, CE12).

Approaches to knowledge dissemination to farmers vary, from a focus on providing technical support for better crop management and productivity to more comprehensive rural extension services that consider socioeconomic and environmental specificities. However, dissemination strategies primarily rely on individual technical visits, where farmers practice specific skills through hands-on learning. Extensionists are evaluated based on the number of visits conducted annually, with quotas often exceeding what is feasible given infrastructure limitations (CO2). Many interviewees highlight that individual visits, while common, are costly and do not always ensure quality service. Group training, learning tours, and farmer field schools were mentioned as complementary but less frequently used strategies in both regions.

Supplementary Information, Table S4 shows quotes from interviewees from the first two stages of data collection related to knowledge epistemologies, institutional purposes, and implementation strategies that prevail across institutions.

### Analyzing and comparing farmers’ knowledge structures to manage CAFS

Table 2 highlights *climate seasonality* as the most mentioned transmitter variable across ICMs in both municipalities, with dry season particularly affecting CAFS in La Paz. *External projects’ support* is another key transmitter variable, noted by six farmers in Belén and eight in La Paz as they initiated CAFS with external assistance. While *training and extension services* influence CAFS management in Belén, *lack of water-related infrastructure* is a major constraint in La Paz.

**Table 2:** Most mentioned transmitter, ordinary and receiver variables in the individual cognitive maps by municipality

Type of variable	Belén	La Paz
Transmitter variables	Rainy season (8) External projects’ support (6) Training and extension services (5)	Dry season (10) Rainy season (10) External projects’ support (8) Lack of water-related infrastructure (7)
Receiver variables	Cacao quality (5) Self-consumption production (5) Sale of dried cacao beans (4) Income (4)	Cacao quality (8) Cacao production (4) Self-consumption production (4) Associativity (4)
Ordinary variables	Fertilization (8) Weed control (7) Cacao drying (7) Pest control (7)	Grafting (9) Pruning (9) Irrigation (9)

Number of ICMs containing the specific variable is indicated in parentheses. For example, rainy season is mentioned in the 8 ICMs from Belén (*n*_Belén_ = 8, *n*_La Paz_ = 10)

For receiver variables, farmers from both municipalities emphasize *cacao quality* and *self-consumption production* are key outputs. Different receiver variables are perceived by the farmers of each respective municipality: while *cacao production* and *associativity* are reported in La Paz, the *sale of dried cacao beans* and *income* are specifically mentioned in Belén.

Concerning ordinary variables, there is no consensus on the most relevant CAFS management practices. Farmers from La Paz prioritize grafting, pruning, and irrigation, whereas farmers from Belén emphasize fertilization, weed and pest control, and cacao drying.

Observing the three types of variables with the highest centrality in the SCMs (Table 3), some differences are identified versus the ICMs and between municipalities. Regarding transmitter variables, some of the most central coincided with the highly mentioned in the ICMs, however *external projects’ support* and *lack of water-related infrastructure* were the most influential external factors to manage CAFS in Belén and La Paz, respectively. While both *dry and rainy season* impact CAFS in La Paz, only the *rainy season* is perceived as critical in Belén. *Aging of farmers* emerges as a key transmitter variable with high centrality in the SCM of Belén.

**Table 3:** Most central variables in the social cognitive maps by municipality

Type of variable	Belén	La Paz
Transmitter variables	External projects’ support (1.75) Rainy season (1.625) Aging of farmers (1.125)	Lack of water-related infrastructure (1.8) Rainy season (1.3) Dry season (1.1)
Receiver variables	Income (1.5) Self-consumption production (1)	Self-consumption production (0.4) Soil protection and restoration (0.3)
Ordinary variables	Cacao production (8.375) Cacao quality (4.125) Pruning (3.25) Training and extension services (3.25) Pest control (3) Fertilization (3)	Cacao production (7.7) Cacao quality (3.8) Pruning (3.8) Grafting (3) Cacao planting (3) Management of other crops (3)

The density of each variable in the SCMs is indicated in parentheses (*n*_Belén de los Andaquíes_ = 8, *n*_La Paz_ = 10)

Among SCM receiver variables, *self-consumption production* is a significant CAFS output in both municipalities. *Income* holds the highest centrality in Belén (1.5). while farmers from La Paz recognize *soil protection and restoration* are perceived as CAFS benefits, although with low centrality.

Ordinary variables, with both incoming and outgoing connections, have higher centrality values. Some transmitter and receiver variables from ICMs became ordinary variables like *cacao quality* and *cacao production* in both municipalities, and, in Belén, *training and extension services*. *Pruning* has a high centrality value in both locations (3.25 in Belén and 3.8 in La Paz). *Pest control* and *fertilization* are perceived as highly central by farmers in Belén, while *grafting* and *cacao planting* are more significant in La Paz.

### Understanding and comparing complexity of farmers’ mental models to manage CAFS

The Supplementary Information (Table S5) provides an overview of the variables mentioned in both municipalities and their frequencies. In Belén, across eight maps, the averages values were 27 ± 4 variables, 36.75 ± 6.52 connections, and connection-to-variable ratio of 1.36 ± 0.15 were found. In La Paz, ten maps averaged 30.7 ± 4 variables, 39.3 ± 5.12 connections, and a ratio of 1.22 ± 0.178 (Table 4). The ICMs in each municipality show consistency, with low standard deviations in the number of variables and connections, similar density values, and no feedback loops, indicating CAFS management complexity, though slightly higher in La Paz.

**Table 4:** Graph theory calculations for individual and social cognitive maps by municipality

Municipality	Belén		La Paz	
	Individual cognitive maps (mean + SD)	Social cognitive maps	Individual cognitive maps (mean + SD)	Social cognitive maps
Number of variables (*N*)	27 ± 3.3	55	30.7 ± 4	57
Number of connections (*C*)	36.75 ± 6.52	156	39.3 ± 5.12	162
Connections to variables (*C*/*N*)	1.36 ± 0.15	2.84	1.28 ± 0,08	2.84
Density	0.05 ± 0.01	0.052	0.04 ± 0.01	0.050
Number of feedback loops	0 ± 0	0	0 ± 0	0
Number of transmitter variables (*T*)	8.13 ± 1.55	14	8.9 ± 2.08	7
Number of receiver variables (*R*)	4.13 ± 1.25	9	5.2 ± 2.3	8
Number of ordinary variables (*O*)	14.75 ± 2.96	32	16.6 ± 1.9	42
Complexity (*R*/*T*)	0.52 ± 0.15	0.643	0.61 ± 0,29	1.14

(*n*_Belén_ = 8, *n*_La Paz_ = 10)

**Fig. 2:**
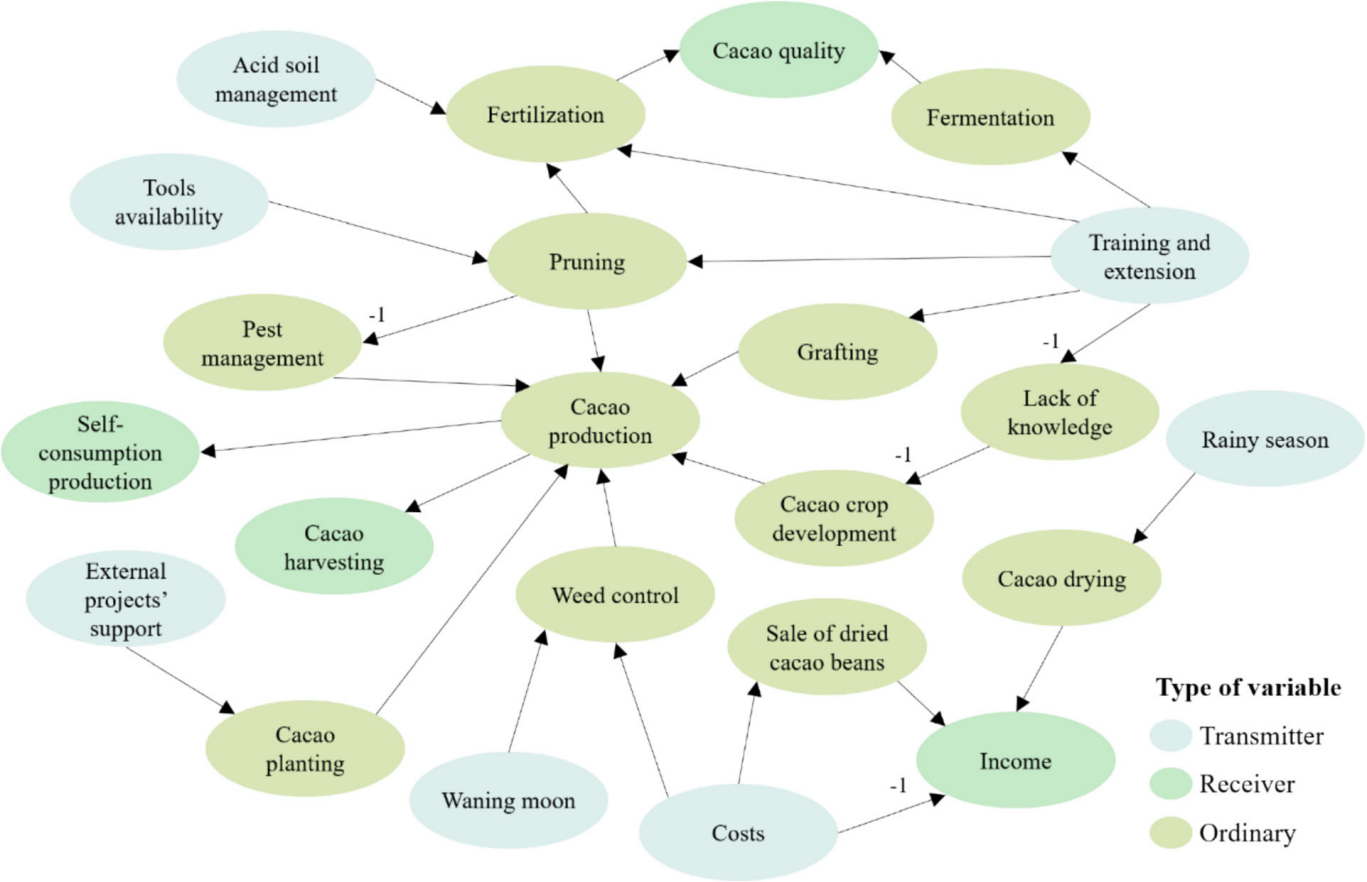
Example of an ICM resulting from interview 1 in Belén. Nodes with text represent variables mentioned by individual farmers about CAFS management on their farms. Directed arrows represent positive relationships between variables and -1 indicates a negative relationship

As for the variables type, more transmitter, receiver, and ordinary variables are identified in La Paz than in Belén on average. In both municipalities, the average number of transmitter variables is higher than receiver variables, leading to complexity ratios below 1. This indicates that farmers perceive more forcing functions than utility outcomes. Figure 2 shows an example of one ICM.

Table 2 shows the SCMs indices from each municipality. A total of 57 variables and 162 connections are identified across farmers in La Paz, while in Belén these are 55 and 156, respectively. Both SCMs have a connection-to-variable of 2.84, with similar density values (0.052 for Belén and 0.050 for La Paz).

SCMs variable types differ between municipalities. In La Paz, the number of receiver variables is greater than the number of transmitter variables, thus a difference compared to the ICMs, reflecting more utility results than forcing functions when pooling farmers knowledge. In Belén, the number of transmitter variables still exceed the number of receiver variables. Mental models complexity for managing CAFS is theoretically higher in La Paz than in Belén (1.14 vs. 0.64, respectively) after adding the variables and connections perceived by all farmers. Additionally, La Paz has more ordinary variables in its SCM than in Belén, indicating a broader perception of CAFS management practices among its farmers.

## Discussion

### CAFS formal knowledge systems in Colombia

CAFS formal knowledge systems are often based on the idea that farmers lack certain knowledge that must be transferred to them (CO2, CO3). While interviews revealed growing recognition of local knowledge for AFS dissemination or employed specific methodologies for incorporating components of farmers’ knowledge systems, some interviewees (e.g., CO8) emphasized the need to transfer missing knowledge (e.g., CO2) and to validate local knowledge through science-based methods. However, such validation processes risk excluding relevant, locally legitimate knowledge, applying inappropriate validation criteria or disempowering communities (Tengö et al. 2014).

Strict registration processes and plant health regulations further limit the dissemination of farmer-selected material, reinforcing power imbalance and knowledge hierarchies, which can lead to counter-productive outcomes (Ferguson et al. 2010), as seen in the Green revolution (Fals-Borda and Mora-Osejo 2003). Conversely, validation can be empowering when designed as a collaborative process, fostering mutual learning and appreciation between knowledge systems (Gratani et al. 2011). This aligns with knowledge co-production, which emphasizes engagement at all stages of knowledge production (Tengö et al. 2014).

Implementation strategies for knowledge production often prioritize field-scale, science-based observations and demonstrations, which may have limited applicability if site-specific differences and changes over time are overlooked. In addition, knowledge dissemination strategies typically emphasize individual technical visits. A practice-based epistemology, centered on social relations and knowledge flows among individual, offers an alternative approach. By integrating farmer observations and experiential knowledge, this approach generate actionable, locally adapted knowledge that informs decision-making (Gardeazabal et al. 2021) while enhancing farmers’ capacity to respond to economic, social, and environmental changes (Lubell et al. 2014).

### Locally situated knowledge for CAFS management

Many differences are observed among the municipalities with respect to the three variable types, showing the need for context-specific thinking. Regarding transmitter variables, CAFS in La Paz are affected by both the rainy and dry season, while in Belén only by the rainy season. This emphasizes the need for two-way learning between knowledge systems to benefit from locally situated knowledge, which is socio-ecologically induced and draws heavily on the available natural resources (Kolawole 2015) and prevailing societal structures.

Our study highlights significant differences in CAFS management practices (ordinary variables) between municipalities, induced by the context-dependent transmitter variables. In Belén, high humidity and regular rainfall make weed control, cacao drying, and pest control priorities, while in La Paz, grafting, pruning, and irrigation are emphasized due to aging plantations and water shortages. Recognizing the precursor biophysical and sociocultural conditions influencing farmers’ decision-making is crucial not only for understanding their perceptions of promoted agricultural practices but also for exposing hidden assumptions often overlooked in top-down approaches (Halbrendt et al. 2014). Since farm management outcomes can create positive or negative feedback that alter local conditions and impact farmer livelihoods (Halbrendt et al. 2014; Kolawole 2015), technical advisory and extension services must be able to address these contextual specificities (e.g., climate seasonality) to enhance CAFS sustainability.

Perceptions of receiver variables or CAFS outputs, both economic (e.g., sales) and non-economic (e.g., self-consumption, soil restoration) vary across case studies, differing from the narratives of researchers and extensionists, which emphasize cacao productivity for increasing farmers’ incomes. However, farmers manage diversified CAFS that provide broader socioeconomic and environmental benefits. Similarly, Averbuch et al. (2022) find that farmers’ motivations differ based on social conditions, with some prioritizing social sustainability outputs and others environmental ones. Cognitive mapping of farmers’ mental models offers a valuable approach to understand situated knowledge regarding goals and values motivating farming behavior.

### Complexity thinking for CAFS management

ICMs show low complexity ratios in both municipalities, suggesting top-down perspectives where forcing functions are well represented, but outcomes remain poorly articulated (Özesmi and Özesmi 2004). Levy et al. (2018) notes that mental models of people involved in sustainable agriculture often follow a hierarchical structure, with few goals and multiple causes. This pattern is evident in the ICMs, where *external projects’ support* emerges as a key transmitter variable in both municipalities. Additionally, all farmers identify climate seasonality variables as forcing functions influencing CAFS outcomes.

La Paz exhibits slightly higher complexity ratios, possibly due to greater experience in managing CAFS (over 20 years), aligning with stratified systems theory (Jaques 1986). However, individual mental models lack complex causal structures, such as bidirectional effects and feedback loops, similar to Levy et al. (2018), who found such structures infrequent even among experienced sustainable agriculture experts.

CAFS, despite being promoted as a sustainable alternative for degraded, deforestation-prone, and conflict-affected areas, farmers’ mental models to manage them reflect hierarchical thinking. Decision-making appears highly influenced by external projects, reliant on external knowledge and shaped by climate variables. This may stem from predominant rationalist knowledge approaches, which prioritize the transfer of prescriptive solutions based on legitimized scientific knowledge over fostering locally perceived options and innovation opportunities (Ferguson et al. 2010).

### Opening-up knowledge systems for CAFS management

Bringing together theoretically farmers’ mental models in each municipality show two potential benefits of activating social learning pathways and connecting knowledge systems. First, it enhances complex reasoning about CAFS, equipping farmers with better tools for management. Second, it shifts perceptions of certain variables from being uncontrollable to manageable. For example, *external projects’ support* shifts from being a transmitter variable (a dependency) to an ordinary variable (a complementary management tool). This implies richer, more democratic maps that have more variables under control and are more adaptable to local environmental changes. Farmers with more democratic mental maps are more likely to see CAFS as changeable, positioning them as active agents in sustainable management (Özesmi and Özesmi 2004). However, previous studies indicated weak bonding ties between farmer associations in the studied regions (Rodríguez et al. 2023). Additionally, when looking at the variables perceived by farmers in the ICMs in both municipalities, *external projects’ support* is mentioned more frequently than *knowledge exchange between farmers*, suggesting a reliance on external interventions over local collaboration.

Although pooling farmers’ knowledge theoretically shows advantages in terms of independence and adaptive capacity, our results suggest that this potential is not being leveraged by knowledge approaches. The high costs of long periods of agronomic evaluation and individual technical visits to farmers could be reoriented to complement technical learning pathways by activating experiential and social learning pathways (Lubell et al. (2014), where local knowledge systems are leveraged (Bonatti et al. 2024). According to Ceccarelli and Grando (2007), approaches that allow for active participation by the target community, like decentralized-participatory plant breeding, result in farming practices that are better suited to the local environment and that empower the community. Future studies can generate more evidence about the impact of experiential and social learning pathways on farmers’ empowerment. Cognitive-based methods appear promising (Biedenweg and Monroe 2013).

## Conclusion

Our characterization of CAFS formal knowledge systems shows a rationalist predominance in institutional purposes and implementation strategies, despite a greater awareness of local knowledge integration. This predominance is reflected by comparatively examining the farmers’ mental models and related knowledge systems, which evidenced hierarchical cognitive structures and simplified cause-and-effect thinking. By theoretically superimposing farmers’ individual mental models, we observed the potential of co-production and social learning to increase the comprehensive understanding of CAFS. Moreover, our results show that CAFS are locally influenced by different forcing functions, motivated by diverse goals and values, while managed with a context-related and environment-induced prioritization of agricultural practices.

Critical examination of our results based on the analytical framework suggests that, although farmers practice CAFS with multiple sustainability benefits, their management is highly dependent on external knowledge and their agricultural systems are highly affected by climate seasonality. We argue that this can be partly explained by predominant rationalist approaches that favors the transfer of prescriptive solutions based on legitimized scientific knowledge. However, the assumptions and blind spots of these approaches affect the outcomes of farming systems and, ultimately, the autonomy and adaptive capacity of farmers. Although the experiential knowledge of farmers results in an improved and more actionable knowledge base to inform locally adapted decision-making, and social learning shows advantages in terms of complex cause-and-effect thinking and local capacity building, this potential is not yet fully tapped by formal knowledge approaches in Colombia.

Our study provides insights about the role of knowledge systems in promoting sustainable agriculture. First, these approaches must build on local knowledge, alongside its contextual embeddedness, employing strategies for knowledge integration, cross-fertilization, and co-production. Second, these approaches require balancing and complementing the dominant technical learning pathway by activating the experimental and social learning pathways. Innovative strategies, which are grounded in an ethic of mutuality, reciprocity and equality between scientific and other knowledge systems, such as participatory prioritization of knowledge needs, cognitive mapping of local knowledge, decentralized and participatory plant breeding and varietal selection, as well as mutual knowledge validation processes can help to integrate context-specific considerations (e.g., climate seasonality, labor availability) in agroecosystem design, thus fostering the emergence of new and more applicable knowledge. In this way, we not only contribute to the endeavor of technically promoting sustainable agriculture, but also to building local capacity and empowering farming communities.

## Supplementary Information

Below is the link to the electronic supplementary material.

Supplementary file1 (PDF 210 KB)

## Data Availability

The data that support the findings of this study are available on request from the corresponding author, Tatiana Rodríguez. The data are not publicly available because it contains information that could compromise the privacy of research participants.
