# Supplementary material for: Rethinking knowledge systems for agroforestry: Insights from the mental models of cacao farmers in Colombia
Source: Ambio. 2025 Apr 30;54(11):1852–66. doi: 10.1007/s13280-025-02189-7 (PMC12480232; doi:10.1007/s13280-025-02189-7)
Supplement: Supplementary file 1 — Supplementary file1 (PDF 210 KB) [file 13280_2025_2189_MOESM1_ESM.pdf]

Ambio - A Journal of Environment and Society

Supplementary Information

This supplementary information has not been peer reviewed.

Title: **Rethinking knowledge systems for agroforestry: Insights from the mental models of cacao farmers in Colombia**

Table S1. Overview of interviewed representatives from research, technical assistance and rural extension institutions supporting cacao sector in Caquetá y Cesar.

Interview code	Institutional role	Department
CA1	Technical assistance and rural extension	Caquetá
CA2	Technical assistance and rural extension	Caquetá
CA3	Technical assistance and rural extension	Caquetá
CA4	Technical assistance and rural extension	Caquetá
CA5	Technical assistance and rural extension	Caquetá
CA6	Technical assistance	Caquetá
CA7	Research, technical assistance and rural extension	Caquetá
CA8	Extension services	Caquetá
CA9	Extension services	Caquetá
CA10	Technical assistance	Caquetá
CA11	Research and technical assistance	Caquetá
CA12	Research and rural extension	Caquetá
CA13	Research and rural extension	Caquetá
CA14	Research	Caquetá
CA15	Technical assistance	Caquetá
CE1	Research, technical assistance and rural extension	Cesar
CE2	Technical assistance	Cesar
CE3	Research, technical assistance and rural extension	Cesar
CE4	Technical assistance and rural extension	Cesar
CE5	Technical assistance and rural extension	Cesar
CE6	Technical assistance and rural extension	Cesar
CE7	Technical assistance and rural extension	Cesar
CE8	Research	Cesar
CE9	Research	Cesar
CE10	Research	Cesar
CE11	Technical assistance	Cesar
CE12	Research	Cesar
CE13	Technical assistance and rural extension	Cesar
CE14	Technical assistance and rural extension	Cesar
CE15	Technical assistance and rural extension	Cesar
CE16	Technical assistance	Cesar
CE17	Technical assistance and rural extension	Cesar
CE18	Research and technical assistance	Cesar

Table S2. Overview of interviewed researchers and extensionists from cacao-related institutions in Colombia.

Interview code	Date	Number of interviewed people	Gender	Role
CO1	14-Apr-21	1	Male	Extensionist
CO2	26-Apr-21	1	Male	Extensionist
CO3	22-Apr-21	1	Male	Researcher
CO4_1<br>CO4_2	30-Apr-21	2	Male<br>Female	Rural extension leaders
CO5	17-Nov-21	1	Female	Research leader
CO6	18-Nov-21	1	Male	Researcher
CO7	18-Nov-21	1	Male	Researcher
CO8	19-Nov-21	1	Female	Research assistant

Table S3. Overview of interviewed CAFS farmers from Belén de los Andaquíes and La Paz.

**Interview code**	**Date**	**Gender**	**Department**	**Municipality**
FCA1	2-Feb-22	Female	Caquetá	Belén de los Andaquíes
FCA2	2-Feb-22	Female	Caquetá	Belén de los Andaquíes
FCA3	2-Feb-22	Male	Caquetá	Belén de los Andaquíes
FCA4	3-Feb-22	Male	Caquetá	Belén de los Andaquíes
FCA5	8-Feb-22	Male	Caquetá	Belén de los Andaquíes
FCA6	8-Feb-22	Male	Caquetá	Belén de los Andaquíes
FCA7	8-Feb-22	Male	Caquetá	Belén de los Andaquíes
FCA8	11-Feb-22	Male	Caquetá	Belén de los Andaquíes
FCE1	17-Feb-22	Female	Cesar	La Paz
FCE2	17-Feb-22	Male	Cesar	La Paz
FCE3	17-Feb-22	Male	Cesar	La Paz
FCE4	17-Feb-22	Female	Cesar	La Paz
FCE5	17-Feb-22	Male	Cesar	La Paz
FCE6	19-Feb-22	Male	Cesar	La Paz
FCE7	19-Feb-22	Male	Cesar	La Paz
FCE8	19-Feb-22	Male	Cesar	La Paz
FCE9	21-Feb-22	Female	Cesar	La Paz
FCE10	21-Feb-22	Male	Cesar	La Paz

Table S4. CAFS research and extension approaches

AFS research and extension approaches	Rationalist approach	Post-rationalist approach
Knowledge epistemologies	<u>*Objectivist view*</u><br>*"... [the farmers] know a lot of things about their farm because they have been there more than we have, but in reality, the technical knowledge, [the extensionists] are the ones who have it" (CO2).*<br><br>*"What we do is to transfer this knowledge to the technical assistants..., who are the ones who will ultimately go to the farm to carry out this technology transfer" (CO3).*<br><br>*"...everything that is found [research outputs] can sometimes be published and is transferred to the technicians..." (CO8).*	<u>*Practice-based view*</u><br>*"I think it is already widely recognized in Colombia that knowledge can come from multiple sources. ..., maybe the challenge is to see how to have a common thread in all of this. But Colombia has already moved beyond discussing the knowledge transfer [as a lineal process] from the expert to the technician, and then to the farmers" (CO4_2).*<br><br>*"...we are working with biological organisms that are very complex, that are subject to edaphic, climatic, and socio-cultural conditions of our farmer..., that cacao variety may be very good, but if the farmer does not like it, everything stops there" (CO6).*<br><br>*"...knowledge is built, and there are conditions and variables that are not controllable. This knowledge must be combined with the local knowledge of the farmers" (CA11).*
Institutional purposes	<u>*Knowledge transfer*</u><br>*"We interact with the farmers very much, and we pass on the knowledge we acquire during the research process" (CO2).*<br><br>*"And what we want is to bring these technological solutions to the farmers so that they can be more productive and competitive" (CO3).*	<u>*Situated learning*</u><br>*"Considering all these arguments that the farmer gives us for the planting: his planting time, way of planting, ... One should never get to impose on the farmer" (CO1).*<br><br>*"What should be promoted is a knowledge dialogue between the empirical knowledge of the farmer and the scientific knowledge of the extensionist, in order to adjust the technical knowledge based on local knowledge..." (CA6).*<br><br>*[Farmers' knowledge] is very important, because [the farmers] also teach us how to adapt the contents, and how they can be better adopted by the farmers (CE6).*
Implementation strategies	<u>*Engineering perspective*</u><br><br>***Research***<br>*"We have been working on [the project of selection, conservation and evaluation of agronomic materials] for many years because it is one of the strong lines of the institution" (CO5).*<br><br>*"We have done evaluation of materials in farmers' plots and.... they can last up to seven years because of the data collection... if we want to register the material, the ICA [Instituto Colombiano Agropecuario] requires a certain amount of time for the agronomic part" (CO8).*<br><br>*"We use transfer activities, such as conferences, symposiums, forums, technical talks, congresses, workshops, meetings, seminars, ..., delivery of planting materials, as well as fairs and visits to the research center" (CO3).*<br><br>***Rural extension & technical assistance***<br>*"...I have within my goals 365 [technical] visits during the year and I have 400 farmers. There are some projects where I have to make four visits to one farmer, ..." (CO2).*<br><br>*"In Colombia we are used to measuring ..., for example, the number of people assisted, and as long as [we] continue to count visits and workshops, [we] will not be able to make much progress" (CO4_2).*	<u>*Emergent perspective*</u><br><br>***Research***<br>*"...this is called participatory varietal selection where the farmer participates with the technician, the trees are selected, they begin to be evaluated... around two or three years... the productive and sanitary part, additionally the compatibility is evaluated..." (CO5).*<br><br>*"... it is key to involve the wisdom of our farmers, because many times what is done is to bring the model that the farmer has, to make a spatial arrangement and monitor its profitability, that is where the success lies when we go hand in hand with our farmers who through their empirical experience know which are the species that have the best allelopathic effects" (CO6).*<br><br>***Rural extension & technical assistance***<br>*"And of course, our trainings are farm to farm, but they are done in different farms each time, so that the farmers..., are exchanging and we are looking at each other's problems" (CO1).*<br><br>*"... we interact and exchange knowledge. I provide [the farmers] with a lot of theory and [the farmers] provide me with some concepts that they also acquire in the field, right? so I think that's the role we play as technicians..." (CO2).*<br><br>*When the service is provided independently, as is the current case, the on-farm visit is the main tool to transmit all [knowledge] to the farmer (CA5).*

Table S5. Overview of variables mentioned by participant farmers in the two municipalities of Colombia.

No.	Variable	Belén de los Andaquíes, Caquetá		La Paz, Cesar	
		Absolute frequency	Relative frequency	Absolute frequency	Relative frequency
1	Associativity	1	13%	8	80%
2	Availability of tools	3	38%	1	10%
3	Bean sorting	1	13%	7	70%
4	Cacao drying	8	100%	8	80%
5	Cacao harvesting	6	75%	10	100%
6	Cacao planting	7	88%	10	100%
7	Cacao pod harvesting	2	25%	0	0%
8	Cacao prices	4	50%	8	80%
9	Cacao production	8	100%	10	100%
10	Sale of wet cacao beans	0	0%	9	90%
11	Cacao quality	8	100%	10	100%
12	Sprouts removal	5	63%	4	40%
13	Care of tree wounds after pruning	0	0%	1	10%
14	Pest control	8	100%	10	100%
15	Costs	6	75%	10	100%
16	Crop growth	3	38%	2	20%
17	Sale of dried cacao beans	7	88%	9	90%
18	Dry season	1	13%	10	100%
19	Efficient use of nutrients	0	0%	1	10%
20	Family integration	1	13%	4	40%
21	Aging of farmers	2	25%	0	0%
22	Cacao fermentation	8	100%	9	90%
23	Fertilization	8	100%	9	90%
24	Floration and pollination	1	13%	2	20%
25	Grafting	6	75%	10	100%
26	Incidence of pests and diseases	2	25%	7	70%
27	Income	4	50%	7	70%
28	External projects' support	6	75%	9	90%
29	Irrigation	0	0%	10	100%
30	Knowledge exchange between farmers	2	25%	5	50%
31	Labor availability	0	0%	1	10%
32	Lack of infrastructure	1	13%	7	70%
33	Lack of knowledge	3	38%	4	40%
34	Production of organic fertilizers	2	25%	2	20%
35	Moon phases	3	38%	1	10%
36	Motivation	3	38%	1	10%
37	Organic certification	2	25%	0	0%
38	Organic residues	5	63%	6	60%
39	Other crops' management	6	75%	10	100%
40	Self-consumption production	5	63%	4	40%
41	Production of plant material	1	13%	3	30%
42	Pruning	8	100%	10	100%
43	Plan material quality	4	50%	3	30%
44	Rainy season	8	100%	10	100%
45	Sales to intermediaries	1	13%	3	30%
46	Shade management	4	50%	5	50%
47	Socio-environmental conflicts	1	13%	2	20%
48	Soil acidity management	5	63%	2	20%
49	Soil protection and restoration	1	13%	2	20%
50	Planting coverage crops	0	0%	2	20%

51	Timber tree planting and regeneration	6	75%	8	80%
52	Topography	3	38%	1	10%
53	Training and extension services	8	100%	7	70%
54	Use of timber trees	0	0%	1	10%
55	Weather conditions	3	38%	0	0%
56	Weed control	8	100%	10	100%
57	Weed growth	5	63%	0	0%
58	Weed management around the tree trunk	2	25%	2	20%
